# Loss of Polycomb Group Protein Pcgf1 Severely Compromises Proper Differentiation of Embryonic Stem Cells

**DOI:** 10.1038/srep46276

**Published:** 2017-04-10

**Authors:** Yun Yan, Wukui Zhao, Yikai Huang, Huan Tong, Yin Xia, Qing Jiang, Jinzhong Qin

**Affiliations:** 1 MOE Key Laboratory of Model Animal for Disease Study, Model Animal Research Center, Nanjing Biomedical Research Institute, Nanjing University, Nanjing, China; 2 School of Biomedical Sciences, The Chinese University of Hong Kong, Hong Kong, China; 3 Department of Sports Medicine and Adult Reconstructive Surgery, Nanjing Drum Tower Hospital Affiliated to Medical School of Nanjing University, Nanjing, China

## Abstract

The Polycomb repressive complex 1 (PRC1) is essential for fate decisions of embryonic stem (ES) cells. Emerging evidence suggests that six major variants of PRC1 complex, defined by the mutually exclusive presence of Pcgf subunit, regulate distinct biological processes, yet very little is known about the mechanism by which each version of PRC1 instructs and maintains cell fate. Here, we disrupted the Pcgf1, also known as Nspc1 and one of six Pcgf paralogs, in mouse ES cells by the CRISPR/Cas9 technology. We showed that although these mutant cells were viable and retained normal self-renewal, they displayed severe defects in differentiation *in vitro*. To gain a better understanding of the role of Pcgf1 in transcriptional control of differentiation, we analysed mRNA profiles from Pcgf1 deficient cells using RNA-seq. Interestingly, we found that Pcgf1 positively regulated expression of essential transcription factors involved in ectoderm and mesoderm differentiation, revealing an unexpected function of Pcgf1 in gene activation during ES cell lineage specification. Chromatin immunoprecipitation experiments demonstrated that Pcgf1 deletion caused a decrease in Ring1B and its associated H2AK119ub1 mark binding to target genes. Altogether, our results suggested an unexpected function of Pcgf1 in gene activation during ES cell maintenance.

Polycomb group (PcG) proteins are known as epigenetic chromatin modifiers that regulate gene expression in multiple cell types and tissue contexts and are critical for cell fate decisions and development^1^,^2^. PcG-mediated gene silencing is associated with specific post-translational histone modifications^3^. Polycomb proteins participate in two major multiprotein complexes: the Polycomb repressive complexes 1 and 2 (PRC1 and PRC2)^4^. PRC2 consists of three core subunits: Eed, Suz12, and the histone methyltransferases Ezh1/2, which is responsible for catalysing both di-methylation and tri-methylation on lysine 27 of the histone H3 (H3K27me3)^5^,^6^. PRC1 mediates the monoubiquitylation of histone H2A at lysine 119 (H2AK119ub1)^7^,^8^. In mammals, all PRC1 complexes contain Ring1A/B which catalyse H2AK119ub1^9^ and Pcgf1-6 which regulates PRC1 enzymatic activity^10^,^11^,^12^. On the basis of the presence or absence of Cbx proteins which can recognize and bind H3K27me3, PRC1 complexes can be grouped as canonical PRC1 and non-canonical PRC1 respectively^13^. Recently, six major groups of PRC1 complexes named PRC1.1–1.6, distinguished by the distinct member of the Pcgf (Polycomb group RING finger protein) family have been reported^14^. Although it is well documented that Polycomb complexes are implicated in stem cell maintenance, very little is known about the functions of each Pcgf family member in ES cells.

Pcgf1 is also known as Nspc1 which is mainly expressed in nervous system^15^. The Pcgf1-containing PRC1 (PRC1.1), also known as the dRing-associated factor (dRAF) complex in D. melanogaster^16^ and the BcoR complex in mammals^17^, has been reported to be important for the deposition of H2AK119ub1. Notably, recent studies revealed that Kdm2b, another component in this complex, plays a critical role in regulating the recruitment of PRC1.1 proteins Ring1B and Pcgf1 to its target genes and most H2AK119 ubiquitylation in ES cells^18^,^19^. Moreover, Knockdown Kdm2b in ES cells led to the failure of proper differentiation^20^,^21^. However, the role of Pcgf1 in regulating H2A monoubiquitylation and pluripotency maintenance in ES cells is not clear.

Here, we use CRISPR/Cas9 strategy to establish Pcgf1 null ES cells. Although the deletion of Pcgf1 does not impair ES cell proliferation and EB formation, mutant cells display profound defects in differentiation. Importantly, RNA-seq analysis reveals that Pcgf1 plays an unexpected role in transcriptional activation, in contrast to the repressive role of canonical PRC1. Mechanistically, this process is initiated by Pcgf1-mediated noncanonical PRC1.1 complex assembly.

## Results

### Pcgf1 knockout ES cell line is established by CRISPR/Cas9 technology

**Figure 1:**
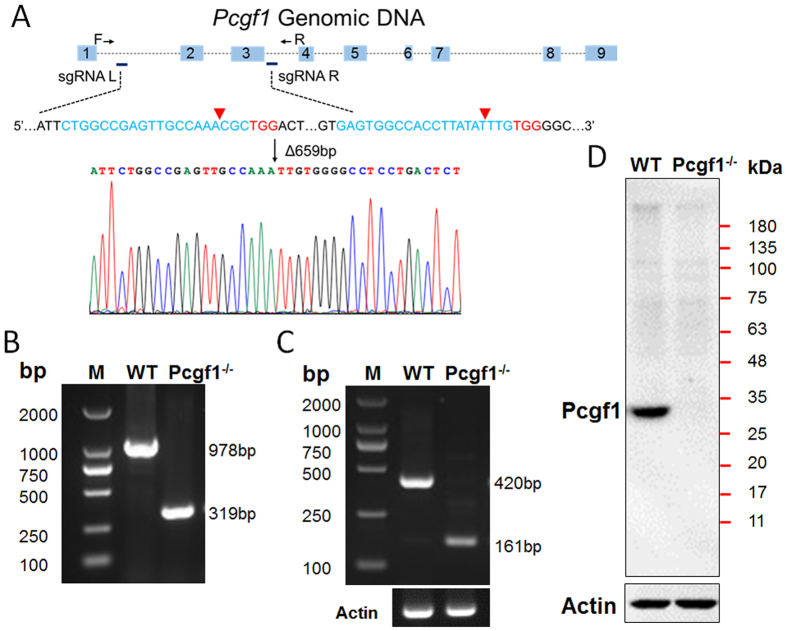
Pcgf1 knockout ES cells are generated by CRSPR/Cas9 technology. (**A**) Schematic diagram of two sgRNAs targeting sites in *Pcgf1*. The sgRNA-targeting sequences were highlighted in blue, and the PAM sequences were labeled in red. PCR products using primers F and R were cloned into plasmid and sequenced. Note that a DNA fragment of 659 bp was deleted. Cas9 mediated a DNA double-stranded breaks ~3 bp upstream of the PAM (red triangle). (**B**) Genotyping analysis of Pcgf1 knockout ES cells (using the F and R primers). (**C**) The *Pcgf1* mRNA level in ES cells was analysed by RT-PCR (M, DNA marker). (**D**) Western blot analysis demonstrated the loss of Pcgf1 protein in ES cells. Pcgf1 was detected as a 30 kDa band in WT extracts but not in extracts from Pcgf1^−/−^ ES cells.

Although it has been reported that PRC1.1 complex is mainly responsible for the H2AK119ub modification in HeLa cells^22^ and NT2 cells^23^, the physiological function of this complex is still not well understood. Recently, study from Barbara Dupret group showed that Pcgf1, the core component of PRC1.1 complex is involved in cell proliferation during early embryogenesis by generating Pcgf1^−/−^ zebra fish^24^. To investigate the role of Pcgf1 in the maintenance and differentiation of mouse ES cells, we established the Pcgf1 knockout ES cell line utilizing the CRISPR/Cas9 genomic editing tool^25^. In order to knockout the region encoding RING finger motif and introduce a frame shift, we designed two sgRNAs specifically targeting exon 2 and exon 3 in the mouse *Pcgf1* gene and cloned them into the PX330 vector which encodes the Cas9 nuclease (Fig. 1A). The two sgRNAs-Cas9 encoding vectors were co-transfected into ES cells. Colonies with successful genome editing were selected by puromycin. To further identify the genomic change of targeting *Pcgf1* by CRISPR/Cas9 system, the genomic DNA of cells was extracted and amplified using the designed primer sets flanking the two cleavage sites by PCR reaction (Fig. 1B) and PCR products covering the target site were confirmed through Sanger sequencing (Fig. 1A). Furthermore, truncated transcript of *Pcgf1* was assessed by reverse transcription and polymerase chain reaction (RT-PCR) amplification. Sanger sequencing of the truncated transcript showed the deletion of 259 bp in *Pcgf1* mRNA and also introduction of a frame shift to the truncated coding sequence (Fig. 1C and Figure S1). To verify whether the *Pcgf1* gene was completely knocked out, we examined Pcgf1 protein expression in the positive clones using Western blot analysis with a monoclonal antibody which specifically recognizes an epitope encoded by exon 4–9 (Fig. 1D). Our results clearly indicated that Pcgf1 protein expression was totally ablated in Pcgf1^−/−^ ES cells and truncated proteins were not detected.

### Pcgf1 is dispensable for ES cell self-renewal

**Figure 2:**
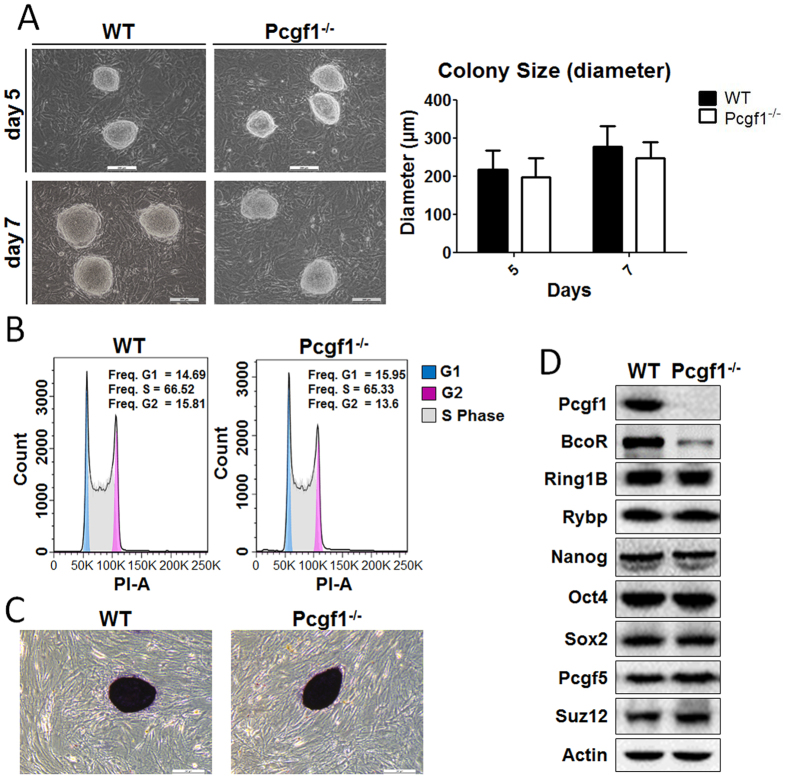
Pcgf1 is dispensable for ES cell self-renewal. (**A**) Representative phase images of WT and Pcgf1^−/−^ ES cell colonies. WT and Pcgf1^−/−^ ES cell colonies were photographed at days 5 and 7 after seeding single-cell suspensions onto MEF-feeder layers. Bar graph showed the mean diameter of 20 random ES cell colonies from three independent experiments. Data are presented as mean ± standard deviation of triplicate experiments. Images were taken at 100× magnification at days 5 and 7. (**B**) Cell cycle distribution of WT and Pcgf1^−/−^ ES cell was analysed by flow cytometry. Top right corner data represented the percentage of cells within the different cell cycle phases. (**C**) The alkaline phosphatase (AP) activity was examined and ES cell colonies were photographed under microscope. Images were taken at 100× magnification at days 7. (**D**) Protein levels were determined by Western blot in WT and Pcgf1^−/−^ ES cells (BcoR, Ring1B and Rybp belong to PRC1.1, Nanog, Oct4, Sox2 are pluripotency markers, Pcgf5 is a component of PRC1.5 and Suz12 is the core subunit of PRC2, Actin was shown as a loading control).

One of the main characteristics of ES cells is self-renewal, which is the capacity to propagate indefinitely while retaining the cellular potential of differentiation into multiple cell types^26^. To elucidate the role of Pcgf1 in the maintenance of ES cell self-renewal, wild-type and Pcgf1^−/−^ ES cells were cultured on mitomycin-C inactivated MEF feeder layer. The Pcgf1^−/−^ ES cells displayed the ability to form ES cell colonies. These colonies exhibited morphology similar to those from wild-type ES cells. Furthermore, Pcgf1^−/−^ ES colony size was comparable to wild-type (Fig. 2A). Consistent with this, we found that ES cells loss of Pcgf1 had no difference in the fraction of cells in G1, G2 and S phase compared to wild-type by using flow cytometry analysis (Fig. 2B). To check pluripotency status, we performed alkaline phosphatase (AP) assay with wild-type and Pcgf1^−/−^ cell colonies on MEF feeder cell layer. Pcgf1^−/−^ ES cells showed high AP activity (Fig. 2C). In agreement with these observations, overall expression levels of ES cell core pluripotency factors (Oct4, Nanog and Sox2) were not significantly altered upon knockout of Pcgf1 (Fig. 2D). Taken together, these results indicated that Pcgf1 was dispensable for self-renewal of mouse ES cells. Western blot analysis also showed the protein levels of other components of PRC1.1 (Ring1B and Rybp), PRC2 member Suz12 as well as other PCGF family member (Pcgf5) were not changed in Pcgf1^−/−^ ES cells (Fig. 2D). Interestingly, Western blot also showed that the expression level of BcoR protein was reduced in Pcgf1^−/−^ ES cells, suggesting that Pcgf1 regulated the stability of BcoR protein (Fig. 2D). This is in agreement with those obtained by other authors who reported that knockdown of Pcgf1 resulted in reduced levels of the BcoR in NT2 cells^23^.

### Pcgf1 is required for ES cell differentiation

**Figure 3:**
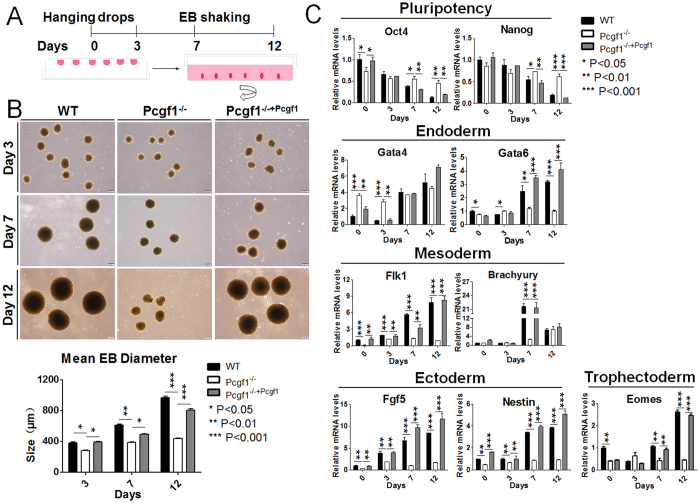
Pcgf1 is essential for ES cell differentiation. (**A**) Schematic representation of Embryoid body (EB) assay. (**B**) Morphological appearance of WT and Pcgf1^−/−^ EBs at days 3, 7 and 12 in suspension culture. Images were taken at 50× magnification. Bar graph showed the mean diameter of 20 random EBs from three independent experiments. Data are presented as mean ± standard deviation of triplicate experiments. (**C**) RT–qPCR was used to measure the expression levels of ES cell lineage-specific markers (endoderm, mesoderm, ectoderm and trophectoderm) and pluripotency markers in WT and Pcgf1^−/−^ EBs on day 0, 3, 7 and 12. Error bars indicated ± SD. Bar graphs represented the mean of three independent biological repeats. *p < 0.05, **p < 0.01, ***p < 0.001 by two-tailed Student’s t test.

Majority of PcG components has been identified as necessary for proper ES cell differentiation^27^. We next examined the differentiation properties of Pcgf1^−/−^ ES cells. We first generated embryoid bodies (EBs) in hanging drops at the first three days and subsequently maintained them in rotating conditions in the absence of LIF (Fig. 3A), and examined EBs morphology by microscopy. EBs mimic, to some extent, early embryonic development and are often utilized as an *in vitro* differentiation assay to test ES cell pluripotency^28^. Our results showed that null ES cells retained the ability to differentiate into EBs. From days 3–12, Pcgf1^−/−^ EBs were macroscopically very similar to wild-type EBs; however, these mutant EBs were smaller than their wild-type counterparts. We randomly chose 20 EBs at 3, 7 or 12-day culture and scored their relative diameters microscopically (Fig. 3B). Our data indicated that Pcgf1^−/−^ ES cells formed EBs with an average size half that of the wild-type. These proliferation defects in the Pcgf1^−/−^ EBs suggested a delayed differentiation of Pcgf1 deficient ES cells. Of note, lentiviral expression of FLAG-tagged Pcgf1 in Pcgf1^−/−^ entirely rescued normal EB size (Fig. 3B). RT-qPCR analysis of 12-day EBs demonstrated that the expression of ES cell marker genes Oct4 and Nanog were dramatically decreased during the process of wild-type EB differentiation (Fig. 3C). Consistent with their aberrant EB formation, however, the Pcgf1^−/−^ EBs maintained high levels of Oct4 and Nanog mRNA over the 12 days of culture, displayed severe misregulation of the differentiation marker genes in comparison to the wild-type. Although all lineage genes were upregulated after EB induction, the mesoderm- and ectoderm-specific genes (Flk1, Brachyury, Fgf5 and Nestin) were markedly downregulated in EBs derived from Pcgf1 knockout ES cells (Fig. 3C). In particular, we detected significant decreased Flk1 expression in undifferentiated Pcgf1^−/−^ ES cells and during EB culture, indicating that Pcgf1 activates Flk1 gene expression which is consistent with our RNA-seq analysis (Supplementary Table 2). Interestingly, the endoderm differentiation might not completely be disturbed in the absence of Pcgf1 because Gata4 was almost normally induced in EBs derived from knockout ES cells (Fig. 3C). It has been reported that Ring1B represses the expression of Gata4 via direct binding to its promoter regions^29^. Moreover, the data in this manuscript demonstrated that Pcgf1 is required for the ordered chromatin recruitment of Ring1B (see the proposed model below). Therefore, we propose that Pcgf1 displays specificity toward endoderm formation through modulating Ring1B activity. Notably, trophectoderm marker Eomes was also reduced in knockout ES-derived EBs (Fig. 3C). Remarkably, lentiviral expression of FLAG-tagged Pcgf1 was able to restore the pattern of expression of differentiation markers to levels similar to those in control cells. Collectively, these data suggest that Pcgf1 deletion impairs ES cell differentiation *in vitro*, likely by preserving expression of high levels of Oct4 and Nanog, which in turn perturb the differentiation process.

### Pcgf1 works as a transcription activator

**Figure 4:**
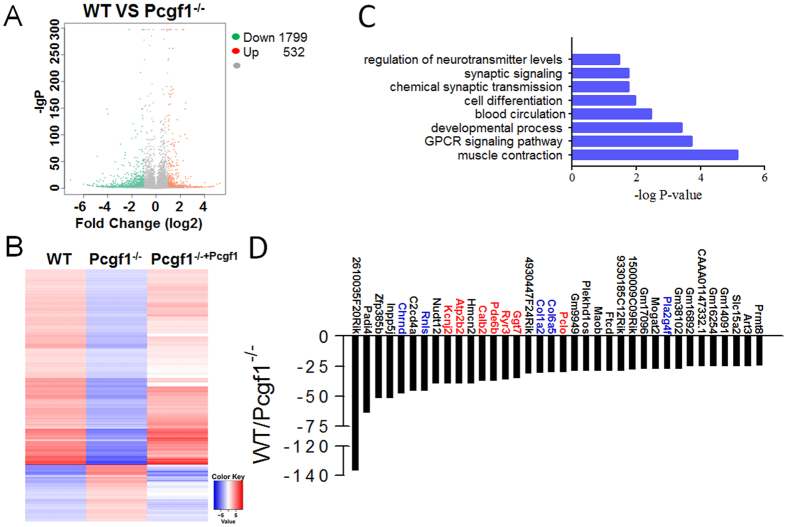
Pcgf1 works as a transcriptional activator. (**A**) Volcano plots represented differentially expressed genes in Pcgf1^−/−^ compared to WT ES cells. Red colour indicated upregulated genes and green colour indicated downregulated genes if they had a log2 fold change of >1 or less than −1, respectively. The differentially expressed genes number were indicated at right. (**B**) A heat map of the 1929 rescuably expressed transcripts with >2-fold expression differences in WT and Pcgf1^−/−^ ES cells. Red indicated high expression and blue indicated low expression. (**C**) GO analysis of biological functions of deregulated genes in Pcgf1^−/−^ ES cells. (**D**) Fold changes in the expression levels of the top 36 downregulated genes, bars showed more than 24-fold change genes. The ectoderm specific genes are highlighted in red and the mesoderm specific genes are highlighted in blue.

To understand the underlying mechanism by which Pcgf1 affects ES cell differentiation, it is critical to characterize Pcgf1 regulated transcripts. To this end, we performed RNA-seq analysis on Pcgf1^−/−^and wild-type ES cells. RNA-seq analysis identified 2331 genes with >2-fold altered expression levels in Pcgf1^−/−^ compared to wild-type ES cells (Fig. 4A, Supplementary Table 2). Importantly, re-expression of Pcgf1-FLAG was accompanied by 82% of these genes altered >2-fold in the opposite direction (Pcgf1-FLAG infected cells compared with Pcgf1^−/−^). Together, these criteria revealed a set of 1929 Pcgf1 target genes. 1491 (77%) genes were downregulated in the absence of Pcgf1 while only 438 genes (23%) were upregulated (Fig. 4B). Expression of some of the transcripts identified as downregulated by RNA-seq analysis was evaluated independently by RT-quantitative PCR (RT-qPCR) (see figure below). The RNA-seq data have been deposited at the Gene Expression Omnibus under accession number GSE95383. Thus, Pcgf1 generally functioned as a transcriptional activator in ES cells. Next, we used gene ontology (GO) analysis to identify the functions of the significantly downregulated genes. These genes were enriched in many functional categories which conformed to the differentiation phenotype we observed, like the development of mesoderm (muscle contraction, blood circulation) and ectoderm (regulation of neurotransmitter levels and synaptic signaling) (Fig. 4C). Figure 4D showed 36 genes downregulated with >24-fold decrease. As expected, these genes were mainly associated with mesoderm and ectoderm differentiation or related to pathways essential for these two germ layer differentiation (e.g. Pla2g4f, Col6a5, Col1a2, Rnls and Chrnd for mesoderm; Pclo, Ryr3, Pde6b, Calb2, Atp2b2 and Kcnj2 for ectoderm). Thus, Pcgf1 acts predominantly as a transcriptional activator which regulates mesoderm and ectoderm differentiation in ES cells.

### Pcgf1 is essential for the recruitment of PRC1.1

**Figure 5:**
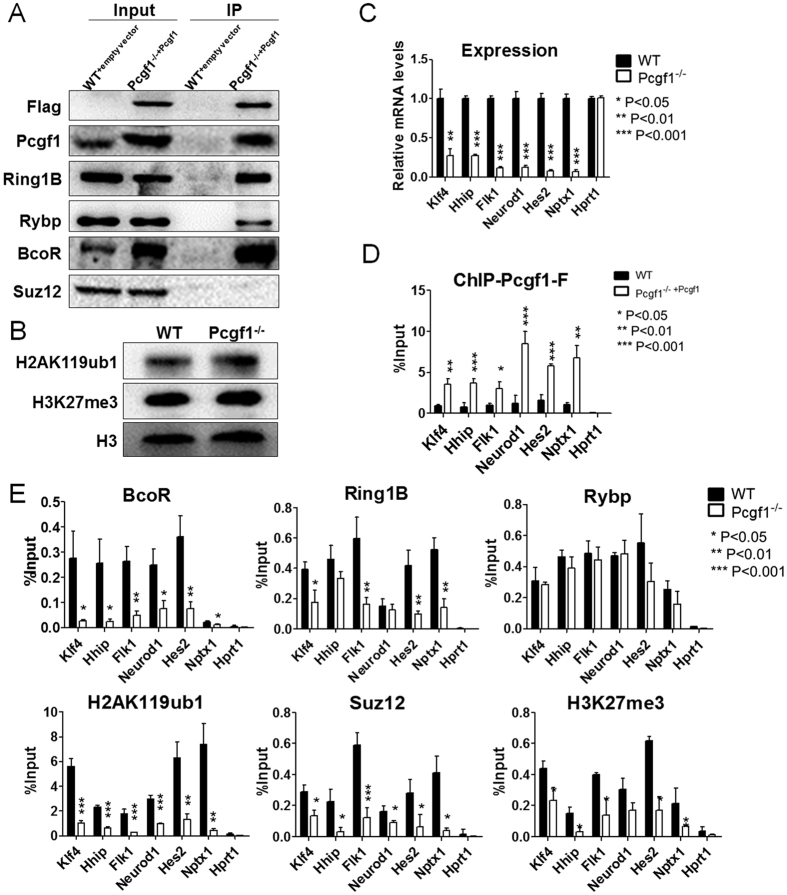
Pcgf1 recruits PRC1.1 in ES cells. (**A**) Immunoprecipitation of PRC1.1 components with anti-Flag antibody in nuclear extracts made from WT cells infected with empty vector (lane 3) or Pcgf1^−/−^ cells rescued with Flag-tagged Pcgf1 (Pcgf1^−/−+Pcgf1^) (lane 4). Antibodies for western blots were indicated to the left. 5% of the total cell lysate used for each immunoprecipitation was loaded in lanes 1 and 2. (**B**) H2AK119ub1 and H3K27me3 levels in Pcgf1^−/−^ ES cells. WT and Pcgf1^−/−^ ES cells nuclear extractions were used to examine H2AK119ub1 and H3K27me3 levels by Western blot, H3 was used as a loading control. (**C**) Expression of six selected target genes (Klf4, Hhip, Flk1, Neurod1, Hes2 and Nptx1) in WT and Pcgf1^−/−^ ES cells. Hprt1 was a negative control. (**D**) Flag ChIP-qPCR analysis was performed in the designated ES cells. (**E**) ChIP-qPCR was used to analyse the occupancy of BcoR, Rybp, Ring1B, H2AK119ub1, Suz12 and H3K27me3 on Pcgf1 targeting genes in WT and Pcgf1^−/−^ ES cells. Error bars indicated ± SD. Bar graphs represented the mean of 3 independent biological repeats. *p < 0.05, **p < 0.01, ***p < 0.001 by two-tailed Student’s t test.

Previous studies demonstrated that Pcgf1 associates with Kdm2b, Rybp, Ring1A/B and BcoR in MEL^30^, HeLa S3 and HEK293 cells^17^ and together they form a noncanonical PRC1 complex in human HEK293T cells^14^, referred to as PRC1.1. To determine that Pcgf1 can indeed associate with PRC1 in ES cells, we performed immunoprecipitation using protein extracts derived from Pcgf1^−/−^ ES cells rescued with Flag-tagged-Pcgf1. We found that Flag-Pcgf1 co-immunoprecipitated with Ring1B, BcoR and Rybp, but not Suz12 (Fig. 5A). This result suggested that Pcgf1 can form a variant PRC1 complex (PRC1.1) in ES cells which consists of Ring1B, BcoR and Rybp, which is consistent with previous findings^14^,^17^,^23^,^30^. Previous study showed Pcgf1 interacts with H2A and enhances H2A ubiquitination *in vivo* and *in vitro*^22^. However, we found global H2AK119ub1 and K3K27me3 levels were unchanged in Pcgf1^−/−^ ES cells compared to wild-type cells. This indicated that the biological functions of Pcgf1 may not depend on the global H2AK119ub1 (Fig. 5B). To explore the local impact of Pcgf1 on chromatin modifications at transcriptional start sites, we selected a group of Pcgf1 target genes (Klf4, Hhip, Flk1, Neurod1, Hes2 and Nptx1) from RNA-seq analysis. In particular, Klf4 is one of four transcription factors in induced pluripotent stem cells (iPS) induction^31^; Flk1 is the earliest known marker of the mesoderm^32^. Hhip^33^, Neurod1^34^, Hes2^35^ and Nptx1^36^ are mainly implicated in controlling ectoderm differentiation or related to pathways essential for ectodermal fate specification. RT-qPCR analysis showed that the expression level of these six transcripts were significantly reduced in Pcgf1^−/−^ ES cells, which was consistent with RNA-seq analysis (Fig. 5C). ChIP-qPCR analysis using Flag antibody in the Pcgf1^−/−^ ES cells rescued with Flag-tagged-Pcgf1 and parental control cells confirmed the specific binding of Pcgf1 at the promoters of these identified targets genes (Fig. 5D). This suggested Pcgf1 regulates its targeting genes by direct binding.

ChIP-qPCR with antibodies directed against each PRC1.1 component (BcoR, Ring1B and Rybp) and PRC2 core subunit Suz12 was performed on these targets. Additionally, ChIP-qPCR was done on the Pcgf1^−/−^ ES cells to determine the effect of Pcgf1 deletion on PRC1.1 and PRC2 occupancy at specific target promoters. PRC1.1 component (BcoR, Ring1B and Rybp) and PRC2 subunit Suz12, as well as their associated H2AK119ub1 and H3K27me3, were enriched at these 6 targets. The enrichment of BcoR, Ring1B and Suz12 were greatly reduced in Pcgf1^−/−^ ES cells. In contrast, deletion of Pcgf1 did not alter the binding of Rybp (Fig. 5E). Notably, the reduction of BcoR occupancy might partially due to the instability of this protein in Pcgf1^−/−^ ES cells as mentioned before. Finally, despite our observation that global H2AK119ub1 and H3K27me3 was not affected by loss of Pcgf1, we observed that enrichment of these two histone modifications were reduced by 3 to 17-fold in the Pcgf1^−/−^ ES cells at these specific targets of Pcgf1. These results are consistent with recent findings that PRC1-dependent H2AK119ub1 is a recruitment cue for PRC2 and H3K27me3^18^,^37^,^38^. Collectively, our results demonstrated that Pcgf1 has a key role in regulating Ring1B recruitment to its target genes in ES cells.

## Discussion

The PRC1 family can be divided into at least six groups, referred to as PRC1.1–1.6, based on the identity of the Pcgf subunit^14^. However, the biological function of each group is still unclear. In this study, we were focused on the Pcgf1 which is a core component of PRC1.1 complex. We generated Pcgf1 gene deficient ES cells by CRISPR-Cas9. We found that Pcgf1 is not essential for the self-renewal of ES cells (Fig. 2). However, Pcgf1 can promote the development of mesoderm and ectoderm during differentiation process *in vitro* (Fig. 3C). Notably, loss of Pcgf1 results in reduced levels of the BcoR protein. Interestingly, it has been reported that BcoR plays a role in the differentiation of ES cells into mesoderm and ectoderm^39^.

Genetic evidence indicate that the role of PRC1 function as a transcriptional repressor through epigenetic mechanisms^6^,^13^. Nonetheless, multiple lines of evidence exist to support a role for PcG in transcriptional activation^40^,^41^. Recently, it has been reported that many unmethylated CpG islands that are targeted by Kdm2b, one component of PRC1.1, are found at the promoters of actively transcribed genes, hinting that this complex could act as transcriptional activators to promote differentiation by targeting early lineage-specific genes in ES cells. Our RNA-seq analysis showed that 1491 target genes were downregulated in Pcgf1^−/−^ ES cells compared to wild-type ES cells, whereas only 438 target genes were upregulated in all 1929 target genes with >2-fold altered expression levels in Pcgf1^−/−^. Therefore, Pcgf1 primarily works as a transcription activator in ES cells (Fig. 4). However, our observations are contradictory to previously published data demonstrating that Pcgf1 represses transcription when fused to the GAL4 DBD in COS-7 cells^42^. Therefore, Pcgf1 might impact transcriptional activity in a cell type-dependent manner. Additionally, Zhonghua Gao *et al*. recently reported that another noncanonical PRC1 complex, PRC1.5, can activate transcription through recruiting CK2 and co-activator P300 by one of its core component, Auts2^40^,^41^. In the future study, we will further explore which component in PRC1.1 complex can recruit transcriptional co-activator to render this complex capable of transcription stimulation.

Recent observation showed that H2AK119ub1 is essential for PRC1 mediated gene repression^19^. Previously, Pcgf1 has been shown to enhance H2AK119ub1 and knock down of Pcgf1 by siRNAs reduces H2A ubiquitinylation level in HeLa cells^22^. In contrast, our study showed that the global level of H2AK119ubl is unchanged in Pcgf1^−/−^ ES cells (Fig. 5B), which is consistent with a recent study in zebra fish^24^. Therefore, the global level of H2AK119ub1 is probably mediated by other component of PRC1.1. Notably, the unchanged levels of H2AK119ub1 in Pcgf1^−/−^ might also be due to a result of redundant and compensatory mechanisms that have evolved to maintain global H2AK119ub1 levels. Experiments examining the effects of combinational deletion mutants of Pcgfs may further clarify the specific roles that different version of PRC1 play in the maintenance of H2AK119ub1 levels. Indeed, knockdown of Kdm2b results in an approximately 40% global reduction of H2AK119ub1^19^. Furthermore, we found that deletion of Pcgf1 resulted in the reduction of Ring1B and its associated histone mark H2AK119ub1 at Pcgf1 target genes. This result is similar to those observed for Pcgf6^−/−^ ES cells, suggesting a common molecular mechanism controlling Polycomb recruitment by Pcgf family in ES cells^43^. Of note, the occupancy of BcoR (another component of PRC1.1) at these targets is also decreased. The reduction of BcoR enrichment in Pcgf1^−/−^ ES cells is probably partially due to the destabilization of its protein level (Fig. 2D). Therefore, Pcgf1 is required for the recruitment of Ring1B and/or BcoR to its target genes (Fig. 5E). Interestingly, the occupancy of PRC2 component Suz12 and its chromatin modification H3K27me3 on Pcgf1 targets are also reduced in Pcgf1^−/−^ ES cells. This is consistent with recent studies which suggest that PRC1-dependent H2AK119ub1 acts as cue for the downstream H3K27me3 deposition by PRC2 complex^18^,^37^,^38^. As mentioned before, Pcgf1 works as a transcription activator in ES cells. The co-localization of Pcgf1 and H2AK119ub1 on Pcgf1 targets suggests that Pcgf1-mediating gene expression in ES cells might be H2AK119ub1-independent.

**Figure 6:**
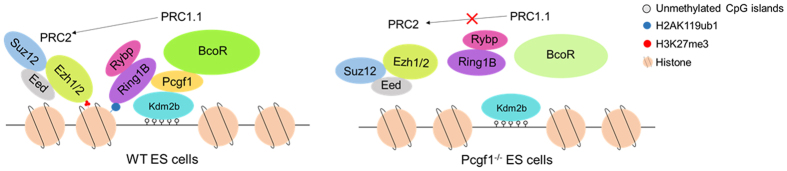
Model of Pcgf1 Function in ES cells. In WT ES cells, Kdm2b interacts with Pcgf1 to recruit the PRC1.1 complex to unmethylated CpG islands. This complex is then capable to catalyse H2AK119ub1 and this histone modification in turn promotes the binding and/or activity of PRC2 complex. In Pcgf1^−/−^ ES cells, disable PRC1.1 could not bind to the chromatin and thus impairs PRC2 complex recruitment.

Although the Pcgf1-deleted ES cells do not show detectable proliferation defects and form EBs with an efficiency similar to that of wild-type cells, mutant cells exhibit severe defects in differentiation *in vitro*. These phenotypes are very similar to the ones recently reported for Kdm2b knockdown ES cells^20^,^44^, suggesting there could be overlapping functions among the PRC1.1 complexes. The failure of Pcgf1^−/−^ ES cells to undergo proper differentiation is consistent with the inability to fully inactivate core pluripotency genes (Oct4 and Nanog) and the absence of transcriptional activation of lineage marker genes during differentiation in the same cells. Accordingly, our results indicate that Pcgf1 and Kdm2b share common biological functions. Moreover, Kdm2b knockdown in ES cells also demonstrates a critical function of Kdm2b in recruiting PRC1 to CpG Islands of developmental regulators^19^,^21^. Future study need to address how Pcgf1 and Kdm2b cooperatively contribute to the PRC1.1 chromatin recruitment and establishment of a specialized chromatin state. Based on these findings, we propose a model for Pcgf1-mediated PRC1.1 chromatin recruitment. We propose that Pcgf1 can interact with Kdm2b which can recognize unmethylated CpG islands and then recruit other components of PRC1.1 to target genes. H2AK119ub1 deposited by this complex, in turn, recruits PRC2 (Fig. 6).

We observed *in vitro* that ES cells deficient in Pcgf1 display severe defects in ectoderm and mesoderm differentiation. Additionally, Pcgf1 has been involved in the proliferation and differentiation of tumor cells^45^. Further studies using Pcgf1 conditional mice will shed light about the *in vivo* contributions of Pcgf1 during early development, homeostasis of the tissue and tumorigenesis.

## Methods

### ES cell culture

ES cells were co-cultured with mitomycin-inactivated murine embryonic fibroblasts (MEFs) on gelatinized tissue culture plates in DMEM (Gibico) supplemented with 15% fetal calf serum (Gibico), non-essential amino acids (Gibico), leukemia inhibitory factor (LIF), penicillin/streptomycin (Sunshine Biotechnology), L-glutamine (Sunshine Biotechnology) and 0.1 mM β-mercaptoethanol (sigma) as described^46^ at 37 °C with 5% CO_2_.

### Generation of Pcgf1^−/−^ ES cells

Pcgf1^−/−^ ES cells were generated by Cas9 technology as described^25^. Briefly, we designed two sgRNAs by using online tool (http://crispr.mit.edu/). SgRNAs were cloned into the pX330-U6-Chimeric-BB-CBh-hSpCas9 (pX330; Addgene plasmid ID 42230) vector. The sgRNA expression constructs were verified by sequencing. SgRNA-Cas9 vectors were co-transfected with a plasmid encoding puromycin (Puro) resistance into ES cells. After 24 hours, ES cells were treated with puromycin for 48 hours and then seeded on MEF feeder to form single colony. The Pcgf1^−/−^ ES cell colonies were identified via DNA-PCR, RT-PCR and Western blot.

### Generation of Pcgf1 expression vector and establishment of a stable Pcgf1^−/−^ rescued (Pcgf1^−/−+Pcgf1^) ES cell line

The Pcgf1 full-length cDNA (NM_197992) was modified by adding N-terminal Flag-tag (DYKDDDDK) sequence into the foward PCR-primer, followed by cloning into pBluescript KS (-). The complete coding sequence was verified by sequencing. The correct inserts were cloned into lentiviral vector^46^. Lentiviral supernatants were produced as described^46^. Briefly, lentivirus was packaged in 293T cells and concentrated lentiviral supernatant was used to infect Pcgf1^−/−^ ES cells with polybrene (Sigma, final concentration of 8 μg/ml). Puromycin was used to screen positive Pcgf1^−/−^ rescued ES cell line and the FLAG-tagged Pcgf1 expression levels were examined by Western Blot. The primers used for PCR are shown in Supplementary Table 1.

### Alkaline phosphatase (AP) staining

ES cell cultures were fixed with 4% PFA (Solarbio) and stained for alkaline phosphatase activity using an Alkaline Phosphatase Stain Kit (Yeasen) according to the manufacturer’s instructions.

### Cell cycle analysis (Flow cytometry)

ES cells were trypsinized, washed three times with PBS, fixed in ice-cold 75% ethanol (drop-wise, while vortexing) for 30 minutes, and stored at −20 °C for at least 4 hours. Subsequently, cells were washed twice with PBS, harvested and incubated for 30 min at 37 °C with RNase A (100 μg/ml, Vazyme, A411-01/02), and stained with the propidium iodine (20 μg /ml) protected from light for 60 min at 37 °C followed by analysis on a FACS LSRFortessa (BD Biosciences) as described^46^.

### Embryoid body (EB) formation and analysis

ES cells were trypsinized and resuspended in medium without LIF^46^. 30 μL (500–1000 cells/drops) was pipetted onto the Petri-dish plate lid, and 10 mL of PBS were placed on a plate to prevent the drops from desiccation. EBs were grown in hanging drops and were cultured for 3 days (37 °C, 5% CO_2_). Three days later, EBs were harvested and cultured on a rotating shaker (37 °C, 5% CO_2_). Fresh medium was replaced every 2 days to avoid medium exhaustion. Total RNA was collected from day 3, 7 and 12 (Trizol, Invitrogen) and analysed by RT-qPCR.

### Global gene expression analysis, RNA preparation and RT-qPCR

Total RNA collected from ES cells and EBs was purified using Trizol (Invitrogen). RNA was reverse transcribed into cDNA with oligodT or random primers using the HiScriptTM 1st Strand cDNA Synthesis Kit (Vazyme Biotech). Quantitative real-time PCR (RT-qPCR) was performed using PowerUp™ SYBR^®^ Green Master Mix (Invitrogen) on a StepOne^TM^ Software v2.3 (Applied Biosystems). The relative expression of genes was analysed based on the 2^−∆∆Ct^ method using the *Actin* gene as a control. The primers used for RT-qPCR are shown in Supplementary Table 1.

### Nuclear extraction, immunoprecipitation, Western blot analysis and histone extraction

ES cells were harvested and lysed in hypotonic lysis buffer (10 mM Tris–Cl pH 8.0, 1 mM KCl, 1.5 mM MgCl_2_ and 0.5 mM β-mercaptoethanol, 10 mg/ml PMSF, Protease Inhibitor Mix (Sigma)). Nuclear Extracts were prepared from nuclei using lysis buffer (20 mM Tris–HCl pH 8.0, 420 mM NaCl, 1.5 mM MgCl_2_, 0.2 mM EDTA, 1% triton-X-100, 25% Glycerol, 0.5 mM β-mercaptoethanol, 10 mg/ml PMSF, Protein inhibitors). Nuclear extracts from the Pcgf1 Flag-tagged ES cells were incubated with M2 agarose beads (A2220, Sigma), in binding buffer (2/3 volume dilution buffer (20 mM Tris–HCl pH 8.0, 0.2 mM EDTA) and 1/3 volume nuclei lysis buffer) overnight at 4 °C. The beads were washed 3 times with washing buffer (20 mM Tris–HCl at pH 8.0, NaCl 450 mM and 0.2 mM EDTA) and subsequently the bound proteins were dissolved in gel loading buffer. Total proteins were separated by SDS-PAGE. The proteins were transferred on polyvinylidene fluoride (PVDF) membrane and the membrane was blocked with 5% (w/v) non-fat milk for one hour at room temperature and then incubated overnight at 4 °C with antibodies against Pcgf1 (sc-515371, Santa Cruz Biotechnology, 1:1,000), Flag (sc-807, Santa Cruz Biotechnology, 1:1,000), BcoR (12107-1-AP, Proteintech, 1:1000), Rybp (sc-374256, Santa Cruz Biotechnology, 1:1,000), Ring1B (09–723, Millipore, 1:1000), Nanog (sc-134218, Santa Cruz Biotechnology, 1:1,000), Oct4 (sc-5297, Santa Cruz Biotechnology, 1:1,000), Sox2 (sc-17320, Santa Cruz Biotechnology, 1:1,000), Pcgf5 (ab201511, Abcam, 1:1000), β-Actin (A01010-1, Abbkine, 1:1000), Suz12 (sc-46264, Santa Cruz Biotechnology, 1:1,000), H2AK119ub1 (#8240, Cell Signaling Technology, 1:1,000), H3K27me3 (#9733, Cell Signaling Technology, 1:1,000), H3 (17168-1-AP, Proteintech, 1:1000). Then, the membrane was incubated with a horseradish peroxidase-conjugated goat anti-mouse lgG-HRP (sc-2005, Santa Cruz Biotechnology, 1:5000), goat anti-rabbit lgG-HRP (sc-2004, Santa Cruz Biotechnology, 1:5000) for 1 h. Chemiluminescence was detected using the ECL blot detection system. Histone Extraction was performed as described^47^.

### RNA-seq

Total RNA was isolated using Trizol reagent (Gibco, 15596–018) according to the manufacturer’s protocol. The preparation of whole RNA-seq libraries and deep sequencing were performed by the Annoroad Gene Technology Corporation (Beijing, PR China). RNA integrity number (RIN) and the concentration were measured using a 2100 RNA Nano 6000 Assay Kit (Agilent Technologies, CA, USA). The mRNA was enriched with Oligo (dT) mRNA magnetic beads. RNA-seq libraries were prepared using 6 bp random primers and libraries were sequenced on the IlluminaHiSeq X-Ten with 150 bp paired-end reads. RNA-seq reads were mapped to the mouse genome (mm10) using TopHat v2.0.12. Reads per Kilobase Millon Mapped Reads (RPKM) were used to quantitatively estimate gene expression values^48^. The final set of the genes were used for differential expression using DEGseq^49^ for the comparison of genes that were upregulated and downregulated in Pcgf1^−/−^ ES cells using the hypergeometric distribution. The RNA-seq data have been deposited at the Gene Expression Omnibus under accession number GSE95383.

### Chromatin Immunoprecipitation Assay (ChIP)

ChIP was performed essentially as described^46^ previously with minor modifications. Briefly, 5 × 10^7^ ES cells were cross-linked with formaldehyde (37%) to a final concentration of 1% for 10 min at room temperature^46^. The reaction was stopped by adding glycine to a final concentration of 0.125 M. Chromatin were sonicated to an average length about 500 bp–1000 bp using a Bioruptor Sonication System (Diagenode). Aliquot of chromatin solution was used as input. ChIP reactions were performed using the following antibodies: BcoR, Rybp, Ring1B, H2AK119ub1, H3K27me3, Suz12 and anti-Flag M2 Affinity GEL. After extensive washes and reverse cross-linking, DNAs were isolated by DNA gel extraction kit (Axygen). ChIP efficiencies were determined by qPCR and the enrichment was calculated as 2^−ΔCt^, where ΔCt = Ct (ChIP) − Ct (Input). The primers used for ChIP-qPCR are shown in Supplementary Table 1.

## Additional Information

**How to cite this article:** Yan, Y. *et al*. Loss of Polycomb Group Protein Pcgf1 Severely Compromises Proper Differentiation of Embryonic Stem Cells. *Sci. Rep.* **7**, 46276; doi: 10.1038/srep46276 (2017).

**Publisher's note:** Springer Nature remains neutral with regard to jurisdictional claims in published maps and institutional affiliations.

## Supplementary Material

Supplementary Information

Supplementary Table 2
