# Supplementary material for: Loss of Polycomb Group Protein Pcgf1 Severely Compromises Proper Differentiation of Embryonic Stem Cells
Source: Sci Rep. 2017 Apr 10;7:46276. doi: 10.1038/srep46276 (PMC5385539; doi:10.1038/srep46276)

**Loss of Polycomb Group Protein Pcgf1 Severely Compromises Proper Differentiation of Embryonic Stem Cells**

**Yun Yan^1^, Wukui Zhao^1^, Yikai Huang^1^, Huan Tong^1^, Yin Xia^2^, Qing Jiang^3^, Jinzhong Qin^1^**

**Supplemental**

**Figure S1. Schematic diagram of sgRNAs targeting at Pcgf1 loci in mRNA level.** Target sequences and sites of cleavage by Cas9 (red triangles) are indicated in the schematic. The PCR products were analyzed by DNA sequencing.Sequences of Pcgf1 exon1 and exon4 conjunction to show the deletion of exon2 and exon3. Note that deletion of exon2 and exon3 (259bp) resulted in frameshift.

Table 1. List of the PCR Primers used in this study

| Gene Symbol | Forward primer | Reverse primer | Product size (bp) | Application |
| --- | --- | --- | --- | --- |
| Pcgf1 | GTCCGTTAGCTGTGTGAACC | CTCTGTCTAAGCCTCGGGAC | 978 | Genomic PCR |
| Fgf5 | TTGCGACCCAGGAGCTTAAT | CTACGCCTCTTTATTGCAGC | 208 | RT-PCR |
| Nestin | AGGTGTCAAGGTCCAGGATG | AAGGAAGCAGACTCAGACCC | 183 | RT-PCR |
| Brachyury | CCAAGGACAGAGAGACGGCT | AGTAGGCATGTTCCAAGGGC | 280 | RT-PCR |
| Flk1 | GCTTGCTCCTTCCTCATCTC | CCATCAGGAAGCCACAAAGC | 262 | RT-PCR |
| Eomes | TGAATGAACCTTCCAAGACTCAGA | GGCTTGAGGCAAAGTGTTGACA | 280 | RT-PCR |
| Gata6 | CCCACTTCTGTGTTCCCAATTG | TTGGTCACGTGGTACAGGCG | 240 | RT-PCR |
| Gata4 | AAACCAGAAAACGGAAGCCC | ATAGTGAGATGACAGCCCGG | 182 | RT-PCR |
| Oct4 | TCTTTCCACCAGGCCCCCGGCTC | TGCGGGCGGACATGGGGAGATCC | 224 | RT-PCR |
| Nanog | CAGGTGTTTGAGGGTAGCTC | CGGTTCATCATGGTACAGTC | 223 | RT-PCR |
| Nptx1 | CTGTCTCGGGTGAACACTCT | CATGTAGTTGGTCCGCAGTG | 186 | RT-PCR |
| Hes2 | GGCCTTTGAATAGCTACCTCG | GTCAGTGAGGGCGAGTCAT | 154 | RT-PCR |
| Klf4 | AAGAGGGGAAGAAGGTCGTG | GGTAGTGCCTGGTCAGTTCA | 199 | RT-PCR |
| Hhip | TAGCACTTCCACTCCTCTGC | TCGGGAAGTCTGGAAAGCAT | 150 | RT-PCR |
| Neurod1 | CGAGGCTCCAGGGTTATGAG | CGTCCTCTTTCTTGTCTGCC | 186 | RT-PCR |
| Hprt1 | TCAGTCAACGGGGGACATAAA | GGGGCTGTACTGCTTAACCAG | 142 | RT-PCR |
| Pcgf1 | CCGACTAATGCTAAATCCAC | CTGTATTGGAGAAGCAAAGG | 132 | RT-PCR |
| β-Actin | AGCCATGTACGTAGCCATCC | CTCTCAGCTGTGGTGGTGAA | 228 | RT-PCR |
| Hhip | CGGTTCCTGCTACTGTCTCA | GTTGGGAAGTGGGAGGAGAA | 119 | ChIP-PCR |
| Neurod1 | GCAACTCGGCTATATAACCCT | ACGATCTCATAACCCTGGAGC | 101 | ChIP-PCR |
| Nptx1 | CCGGTCCGCACACAATTAAA | TTCTACGCCGCACAGTCTT | 151 | ChIP-PCR |
| Hes2 | CACCCTGTGCAGTGACAAAG | CAGAGAGAAAACCGAAGCCG | 176 | ChIP-PCR |
| Klf4 | GGACCTACTTATCTGCCTTGC | GAGCCCCAAAGTCAACGAAG | 200 | ChIP-PCR |
| Hprt1 | GAATCCTCTGGGAGACGACA | CGGAAAGCAGTGAGGTAAGC | 272 | ChIP-PCR |
| Flk1 | TGTGTTTCCTTAGATCGCGC | CAAAGAAGTCACAGAGGCGG | 193 | ChIP-PCR |


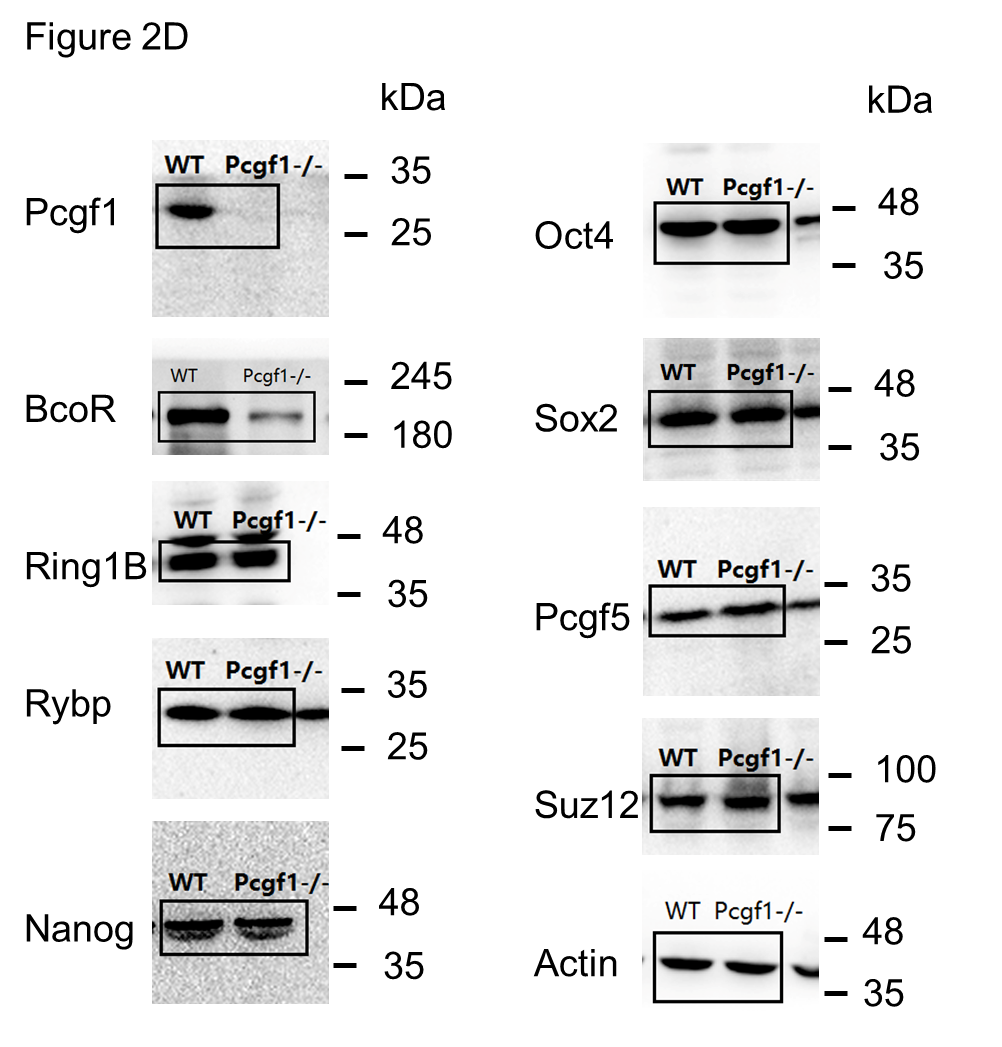

Supplement: Supplementary Information [file srep46276-s1.doc]
